# A case report of multiple endocrine neoplasia type 2B

**DOI:** 10.1097/MS9.0000000000001867

**Published:** 2024-04-03

**Authors:** Mohammad Bagher Jahantab, Babak Rastegar, Arash Aria

**Affiliations:** Department of General Surgery, Shahid Beheshti Hospital, Yasuj University of Medical Sciences, Yasuj, Iran

**Keywords:** case report, medullary thyroid carcinoma, multiple endocrine neoplasia type 2, pheochromocytoma

## Abstract

**Introduction and importance:**

Multiple Endocrine Neoplasia Type 2 (MEN2) is a rare autosomal dominant neoplastic syndrome resulting from RET gene mutations, marked by medullary thyroid carcinoma (MTC) and increased risk of other endocrine tumors. MEN2 includes subtypes MEN2A, MEN2B, and familial MTC. Prophylactic thyroidectomy is recommended for MEN2A due to high MTC risk.

**Case presentation:**

A 38-year-old woman with a family history of thyroid cancer presented with headaches, sweating, and palpable breast mass. Exam revealed skin lesions. Lab abnormalities and imaging indicated a large adrenal mass and thyroid nodules. Inconclusive biopsies led to left adrenalectomy, confirming pheochromocytoma. Subsequent total thyroidectomy revealed MTC.

**Clinical discussion:**

This case represents rare MEN2B presentation, featuring MTC, pheochromocytoma, mucosal neuromas, and marfanoid habitus. Genetic testing for RET mutations is crucial with a positive family history. MEN2A individuals undergo prophylactic thyroidectomy due to high MTC risk. Although rare, pheochromocytoma can be an initial MEN2 manifestation, indicated by paroxysmal symptoms. Surgical resection is the treatment.

**Conclusions:**

The patient’s successful adrenalectomy followed by total thyroidectomy confirmed MTC. Thorough evaluation, including inconclusive initial findings, emphasizes imaging, and biopsies. Early detection and appropriate management optimize MEN2 outcomes.

## Introduction

Highlights

- Rare Presentation of Multiple Endocrine Neoplasia Type 2B: The case report describes a rare presentation of Multiple Endocrine Neoplasia Type 2B (MEN2B), a rare neoplastic syndrome characterized by medullary thyroid carcinoma (MTC) and pheochromocytoma. The rarity of this presentation adds to the existing literature on MEN2B and expands our understanding of the clinical manifestations associated with this condition.
- Importance of Family History: The case report emphasizes the significance of considering a patient’s family history when evaluating for MEN2B. The patient in this case had a positive family history of thyroid cancer and adrenal mass, which raised suspicion for an inherited neoplastic syndrome. This highlights the importance of thorough family history assessment in the diagnosis and management of MEN2B.
- Diagnostic Challenges and Surgical Interventions: The manuscript discusses the challenges encountered in diagnosing and managing MEN2B in the presented case. It outlines the various diagnostic modalities employed, including laboratory testing, imaging studies, and fine needle aspiration (FNA). Additionally, it details the surgical interventions performed, such as transperitoneal open total left adrenalectomy and total thyroidectomy, which led to the final diagnoses of pheochromocytoma and medullary thyroid carcinoma, respectively.
- Prophylactic Thyroidectomy and Patient Outcomes: The case report highlights the importance of prophylactic thyroidectomy in individuals with MEN2A due to the high risk of developing MTC. It underscores the significant morbidity and mortality associated with undiagnosed or inappropriately treated MTC in MEN2B. The successful outcomes and unremarkable postoperative course in our patient emphasize the potential benefits of timely surgical intervention.

Multiple endocrine neoplasia type 2 (MEN2) is a rare neoplastic syndrome inherited in an autosomal dominant fashion consisting of MEN2A, MEN2B, and familial medullary thyroid carcinoma (MTC). MEN2 is characterized by the 100% prevalence of MTC and an increased risk of developing other specific tumors affecting additional glands of the endocrine system due to mutations in the RET gene, located on chromosome 10q11.2^1^. Major characteristics of MEN2A include MTC, pheochromocytoma, and hyperparathyroidism. MEN2B is characterized by MTC, pheochromocytoma, multiple mucosal neuromas, and often a marfanoid habitus^2^. This mutation leads to alterations of C cells derived from the neural crest^3^. In patients with MEN2A syndrome, prophylactic thyroidectomy is recommended due to carriers having a 100% risk of developing MTC during their lifetime^4^. MEN2A represents 95% of all MEN2 while MEN2B represents 5% of the remaining cases^5^. MTC leads to fatal outcomes in MEN2B if it is inappropriately treated or goes undiagnosed. In familial cases where the genetic diagnosis is made with RET mutation gene, a prophylactic thyroidectomy significantly reduces morbidity and mortality^6^.

Pheochromocytoma is rarely the first manifestation of MEN2 and is often identified during screening exams in patients with MEN2.

Pheochromocytoma occurs in 40% of patients with MEN2 and is usually evident about 10 years later after MTC or C cell hyperplasia^7^.

Classically, patients present paroxysmal episodes including headaches, profuse sweating, palpitations and HTN, and therefore tumor resection remains the gold standard of treatment^8^. Most pheochromocytomas are sporadic and unilateral^9^.

## Presentation of the case

**Figure 1:**
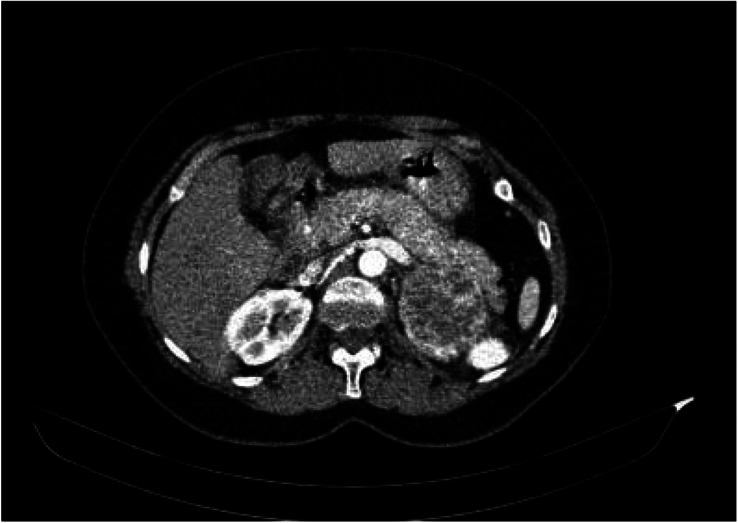
Abdominopelvic CT scan showed large, heterogeneous, enhanced, well-defined solid mass structure with a central necrotic component measuring about 64×52×68, seen on the left side of the adrenal gland.

On 20 January 2023, a 38-year-old female came to the emergency department (ED) with complaints of headaches and sweating. Her blood pressure (BP) was 160/90 mmHg. At that time, her BP was managed, and she was discharged with antihypertension treatment. After unsuccessful treatment of hypertension, she returned with complaints of palpitations, flushing, and sweating. At this time, she claimed that her mother died due to thyroid cancer, and her sister had an adrenal mass at the age of 32. A physical examination showed a palpable mass at the right breast and skin lesions above both breasts, abdomen, and neck. The initial laboratory testing is listed in Table 1. Then the patient underwent abdomenopelvic computed tomography, in which there was evidence of a large, heterogeneous, enhanced, well-defined solid mass structure with a central necrotic component measuring about 64×52×68, seen on the left side of the adrenal gland. The other findings were unremarkable (Fig. 1).

**Table 1:** Initial laboratory tests

		Reference range
TSH	3.79	0.39–6.16
CEA	12.9	<5.5
CA19-9	110	0–39
BUN	9	5–24
Urine 24 h creatinine	668	15–20
Urine 24 h metanephrine	1779	<350
Urine 24 h nmetanephrine	4659	<600
Urine volume	1600	800–2000
Urine 24 h VMA	5.0	0–13.6
Urine 24 h free cortisol	72.6	4.3–176
Aldosterone	125.6	40–310
DHEA-SO4	0.8	0.03–5.88
Testosterone	0.4	0.2–0.95
ACTH	10.52	9–52
Cortisole	26,8	4.3–22.40
Antinuclear antibody	0,17	0–1.2
Anti-ds-DNA	5,3	<16
Anti ss-a native/RO	1.1	<20
Anti ss-b/LA	0.1	<20

Due to her positive family history, neck ultrasonography was done for her, which showed a hypoecho nodule with calcification measuring about 14×13 mm in the right lobe and two hypoecho nodules with calcification measuring about 14×13 mm and 8×7 mm in the left lobe. And a few benign-looking reactive lymph nodes are seen on the bilateral side of the neck, where the largest submandibular region was RT: 16×6 mm and LT: 16×6 mm. Bilateral submandibular glands show normal size, shape, and echopattern. Fine niddle aspiration (FNA) was performed, and the cytopathology report showed atypia of undetermined significance (AUS).

A skin lesion biopsy was taken, whose pathology was skin and subcutis tissue with fibrosis, mild chronic inflammation, and foci of hemorrhage. No evidence of malignancy was seen in these sections examined.

Bilateral breast and bilateral axillary sonography were done for the patient, which showed a murally calcified cyst measuring about 9.4 mm in the inferior aspect of the right breast. Both axillary fossae are intact without mass or lymphadenopathy.

**Figure 2:**
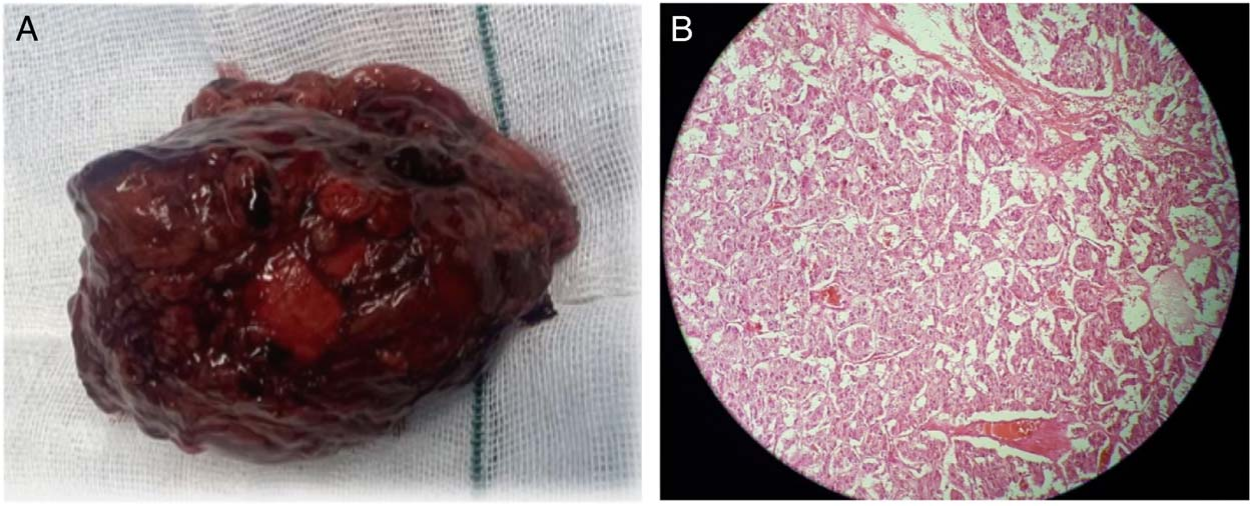
Adrenal gland (A), Nested (zellballen) arrangement with large polygonal cells and a bandant cytoplasm (B).

The patient was prepared for surgery and was given an alpha blocker (Prazosin) to normalize blood pressure. And a transperitoneal open total left adrenalectomy was successfully performed; histologic examination revealed lobulated mass, M: 7.0×7.0 cm. The external surface is smooth and creamy brown. The cut section is encapsulated with a creamy yellow surface. The capsule is completely intact. The final diagnosis was pheochromocytoma. The postoperative course was unremarkable (Fig. 2).

**Figure 3:**
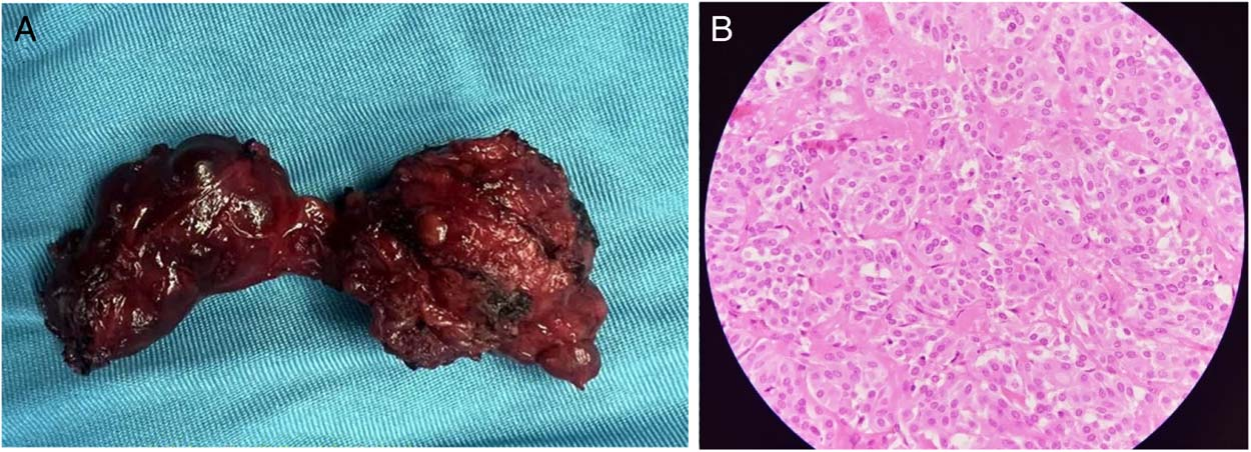
Thyroid gland (A), round and polygonal cells in nest with Round nuclei with finely stippled to coarsely clumped chromatin eosinophilic to amphophilic granular cytoplasm stroma has amyloid deposits (B).

Despite the AUS pathology, the patient had an elevated CA19-9. Two months later, the patient was a candidate for total thyroidectomy, whose pathology was: a total thyroidectomy specimen, including the right lobe measuring 4×2×1.5 cm, a cut section showing a creamy yellow mass measuring 2×1.5×1 cm, the left lobe measuring 3.5×2×1.5 cm, a cut section showing a creamy yellowish mass measuring 2×1×0.8 cm, and the isthmus lobe measuring 2×2×0.5 cm. The final diagnosis was medullary thyroid carcinoma (Fig. 3).

## Discussion

MEN2 is a rare hereditary disease characterized by the presence of MTC, unilateral or bilateral pheochromocytoma, and primary parathyroid hyperplasia^10^ The lifetime penetrance of MTC is nearly 100%, and there is variability in the other manifestations^5^. Patients should be properly worked up when one of the syndromes is identified for MEN2 and family history should also be taken into account. The American Thyroid Association guidelines recommend that prophylactic lateral neck dissections may be considered based on serum calcitonin levels. Prophylactic ipsilateral central and lateral neck dissection should be considered for patients with basal serum calcitonin 20 pg/ml, and contralateral lateral neck dissection should be considered for serum calcitonin >200 pg/ml^11^. It is usually diagnosed in the 2nd–3rd decade, significantly younger than in the sporadic cases^2^. Nowadays, cortical-sparing adrenalectomy has been the preferred approach for patients at risk for, or diagnosed with bilateral pheochromocytoma especially those presenting a predisposing genetic mutation^12^.

## Conclusion

In conclusion, this case report highlights a rare presentation of Multiple Endocrine Neoplasia Type 2B (MEN2B), characterized by the occurrence of medullary thyroid carcinoma (MTC) and pheochromocytoma, along with multiple mucosal neuromas and a marfanoid habitus. MEN2 is an autosomal dominant neoplastic syndrome associated with RET gene mutations. Prophylactic thyroidectomy is crucial for individuals with MEN2A due to the high risk of developing MTC.

## Methods

The work has been reported in line with the SCARE 2023 criteria^13^.

## Ethical approval

None.

## Consent

Written informed consent was obtained from the patient for publication and any accompanying images. A copy of the written consent is available for review by the Editor-in-Chief of this journal on request.

## Sources of funding

None.

## Author contribution

Concept: [MBJ, BR] conceived and designed the study. Design: [AA, MBJ] designed the study. Literature search: [MBJ, BR] conducted the literature search. Data acquisition: [MBJ, BR] collected the data. Manuscript preparation: [MBJ, BR] wrote the manuscript. Manuscript editing: [MBJ, BR, AA] edited the manuscript.

## Conflicts of interest disclosure

None.

## Research registration unique identifying number (UIN)

None.

## Guarantor

Mohammad Bagher Jahantab.

## Availability of data and materials

The data are available with the correspondence author and can be reached on request.

## Provenance and peer review

None.
